# Profile of pediatric patients with myiasis treated at a tertiary hospital in Rio de Janeiro^☆^^☆☆^

**DOI:** 10.1016/j.abd.2020.05.018

**Published:** 2021-03-20

**Authors:** Felipe Tavares Rodrigues, Antonio Macedo D’Acri, Claudia Soares Santos Lessa, Valéria Magalhães Aguiar

**Affiliations:** a Universidade Federal do Estado do Rio de Janeiro, Rio de Janeiro, RJ, Brazil; b Service of Dermatology, Hospital Universitário Gaffrée e Guinle, Escola de Medicina e Cirurgia, Universidade Federal do Estado do Rio de Janeiro, Rio de Janeiro, RJ, Brazil; c Departament of Microbiology and Parasitology, Instituto Biomédico, Universidade Federal do Estado do Rio de Janeiro, Rio de Janeiro, RJ, Brazil

Dear Editor,

Myiasis is a dermatozoonosis, defined by the infestation of living animal tissues (mammals, birds, reptiles, and amphibians) by fly larvae, which can deposit their eggs in the natural orifices of their hosts, in skin continuity solutions or healthy skin, in furuncular myiasis. The larvae grow and feed on the host's tissue, causing severe pain and tissue destruction. This is a neglected disease, resulting from low socioeconomic conditions and the delay in seeking assistance.^1^, ^2^ Surveys on the epidemiological profile of pediatric patients with myiasis are scarce in the literature.

This is an observational study carried out at Hospital Federal do Andaraí (HFA), in the city of Rio de Janeiro, State of Rio de Janeiro, Brazil, where patients aged up to 12 years with myiasis were selected and treated from 2007 to 2015. Socioeconomic and clinical data were collected from the patients' medical record, the larvae were extracted and identified according to taxonomic keys, in the Diptera Study Laboratory of the Federal University of the State of Rio de Janeiro – UNIRIO, as well as the adult insects preserved for around 10 days in sterile material.^3^, ^4^ The study was approved by the UNIRIO Ethics Committee and the HFA Study Center.

A total of 69 patients were evaluated, aged less than or up to 12 years, in the studied period, representing about 19% of the 368 cases treated in all age groups. Most patients were females, 58 (84%), and 47 (68%) were dark-skinned. The family income was mostly up to 2 minimum wages and no head of the family had finished higher education. Only 48 (70%) had access to running water at home, 37 (54%) had access to regular garbage collection services and 41 (62%) had sanitary sewage collection system at the household, of which 18 (44%) had no access to a public sewage system and disposed of the sewage into a nearby pit. Seven (10%) were in a situation of vulnerability (Table 1 and Fig. 1) and only 21 (30%) of the treated children were enrolled in school or daycare centers during the treatment period. The distribution by age group was balanced, with a mean age of 5.89 ± 3.38 years, with a lower prevalence in children under 1 year.

**Table 1:** Socioeconomic and clinical characteristics of pediatric patients with myiasis treated at the Andaraí Federal Hospital between 2007 and 2015.

Characteristics		n	%
Socioeconomic			
Self-declared skin color	White	8	12
	Black	30	43
	Brown	17	25
	Unknown	14	20
Average monthly family income (minimum-wages between R$380 and R$788; 2007–2015)			
None		7	10
Up to 2 minimum wages		37	54
Between 2 and 4 minimum wages		11	16
More than six minimum wages		1	1
Unknown		13	19
Maternal level of schooling			
Illiterate		15	22
Incomplete Elementary School		23	33
Complete Elementary School		10	15
Incomplete High School		3	4
Complete High School		6	9
Incomplete Higher Education		1	1
Unknown		11	16
Regular garbage collection service or appropriate disposal pit close to home			
Yes		37	54
No		27	39
Unknown		5	7
Running water at the household			
Yes		48	70
No		16	23
Unknown		5	7
Sewage collection system at the household			
Yes		41	62
No		22	35
Unknown		6	3
House occupancy status			
Owned		44	64
Rented		17	25
Homeless person		3	4
Living in a shelter		4	6
Unknown		1	1
Clinical information			
Predisposing factors and associated comorbidities			
Bacterial infection (impetigo, cellulitis)		19	27
Trauma		7	10
Pediculosis		26	38
Scabies		4	6
Chemical dermatitis		4	6
Allergic dermatitis (including scrofulous prurigo)		25	36
Seborrheic dermatitis		5	7
Atopic dermatitis		4	6
Affected body sites			
Parietal scalp		32	46
Temporal scalp		8	12
Frontal scalp		4	6
Occipital scalp		19	28
Umbilical scar		1	1
Auricle		1	1
Lower limb		2	3
Number of lesions			
Average		1.8	–
One		42	61
Two		8	12
Three		14	20
Five or more		5	7

**Figure 1:**
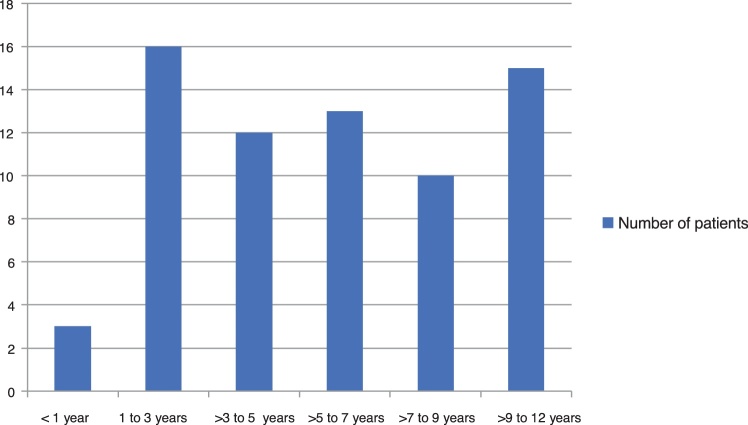
Distribution of myiasis cases in the pediatric population by age group, treated at Hospital Federal do Andaraí between 2007 and 2015.

The cases originated mainly in the North region of the city of Rio de Janeiro, with 42 (61%) of the cases, with emphasis on the regions of great Tijuca and great Méier, which accounted for 22 (52%) of all cases in the North region of the city, the geographical area where the Andaraí Federal Hospital is located. In this same region, 4 (6%) lived in shelters or orphanages and 3 (4%) were homeless in neighborhoods adjacent to the hospital; one of these patient’s entire family had lived for years in an abandoned milk factory. Two (3%) lived in the downtown region and 18 (26%) lived in Baixada Fluminense.

The scalp was the preferred site in 63 (91%) of the children. Among the predisposing factors and associated diseases, other dermatozoonoses represented by pediculosis and scabies, allergic dermatitis, and pyoderma were observed as the most prevalent ones (Table 1). The maximum number of larvae removed from a patient was 36 and the minimum was only one, with an average of 11.5 larvae per patient.

Cavitary or necrobiontophagous myiasis, those associated with pre-existing wounds and low socioeconomic status and poor hygiene conditions, were observed in 58 patients (91%), with *Cochliomyia hominivorax* being the most prevalent species, present in 56 (87%) patients, *Cochliomyia macellaria* larvae were found in three patients (5%), *Chrysomya putoria* in one (1%), *Chrysomya albiceps* in one (1%) and *Musca domestica* in one patient (1%). There were three cases of co-infection by two of these species.

Six (9%) cases of furuncular myiasis were caused by the species *Dermatobia hominis*, a primary disease of cattle and horses that can occur in the general population exposed to the risk in rural and even in urban areas, which does not fit the poverty profile.^1^, ^2^, ^5^, ^6^ There was one case of concomitant infestation of father and daughter, raising the possibility of family co-infection. In some cases, the myiasis occurred under pre-existing conditions: four cases of contact dermatitis due to primary irritant resulting from the use of guanidine, a substance used for hair straightening.

Moreover, there was an association with a thermal burn wound (one case), cutaneous varicella lesion (one case), as well as a tinea capitis lesion (one case).

Although manual extraction is the treatment of choice, ivermectin is widely used in extensive lesions, when it is not possible to remove all larvae, but it should be avoided in children under 5 years of age, according to the medication package insert. Sixteen (23%) of the cases required hospitalization, mainly due to the social vulnerability that made home treatment unfeasible.

A total of 61 patients (88%) received antibiotic therapy with Cephalexin or Amoxicillin to prevent and/or treat secondary bacterial infections. Regarding the prevalence of myiasis cases in the pediatric population, compared to the involvement in all age groups, the present study showed a lower percentage than that found in the literature.^7^, ^8^

The main associated factors for larva infestation in children and adolescents are the presence of other infectious and allergic diseases, with emphasis on pediculosis and lesions caused by scrofulous prurigo, with the act of scratching being the main mechanism for the formation of wounds unlike the adult population, in which wound formation is associated with chronic diseases or diabetic foot ulcers, vascular ulcers, in addition to neoplastic wounds, and where carcinomas are the main foci of infestation.

What is also noteworthy is the change in the main infection site, which in pediatric patients is preferentially the cephalic segment, facilitated by pediculosis, traumas and because it is a region not covered by clothing; on the other hand, in adults, the preferred place is the distal third of the lower limbs.^7^, ^8^, ^9^

This is a problem, except for cases of furuncular myiasis, which mostly affects populations with greater social vulnerability and with poor hygiene conditions and can be prevented with the advance of basic sanitation coverage and public awareness.

## Financial support

Financial support provided by 10.13039/501100003593CNPq - Conselho Nacional de Desenvolvimento Científico e Tecnológico.

## Authors’ contributions

Felipe Tavares Rodrigues: Study design, data collection and analysis, and review of the manuscript.

Antonio Macedo D’Acri: Critical review of the manuscript.

Claudia Soares Santos Lessa: Data analysis and critical review.

Valéria Magalhães Aguiar: Data analysis and critical review.

## Conflicts of interest

None declared.
